# Effects of Particle Size and Replacement Ratio of Ceramsite on Permeability Characteristics of Lightweight Concrete via Pore Structure and Fractal Approach

**DOI:** 10.3390/ma19112305

**Published:** 2026-05-29

**Authors:** Zhe Liu, Yinshan Xu, Shenghan Zhuang, Jiaolong Ren

**Affiliations:** 1 Zhejiang Highway and Waterway Engineering Consulting Co., Ltd., Hangzhou 310013, China; liuzhe_hz@126.com; 2 Zhejiang Scientific Research Institute of Transport, Hangzhou 310023, China; 3 Department of Bridge Engineering, Southwest Jiaotong University, Chengdu 610031, China; shenghanzhuang@my.swjtu.edu.cn; 4 School of Civil Engineering and Geomatics, Shandong University of Technology, Zibo 255000, China

**Keywords:** lightweight concrete, fractal approach, permeability, pore structure, ceramsite

## Abstract

The variation law and mechanism of the permeability characteristics of coal gangue ceramsite lightweight aggregate concrete (CLAC) remain unclear. Therefore, in this study, the effect of the ceramsite size and replacement ratio on the pore structure characteristics of the CLAC was analyzed by mercury pressure test. Moreover, based on a fractal approach, the relationship between permeability characteristics and pore structure of the CLAC was established. The results indicate that incorporating coal gangue ceramsite effectively decreases the maximum pore size. The fractal dimension increases as the replacement ratio of 20–30 mm and 10–20 mm ceramsite rises, whereas an opposite trend is observed when the content of 5–10 mm ceramsite increases. At moderate replacement levels, the introduction of ceramsite aggregates can reduce the fraction of detrimental pores and promote the formation of harmless and slightly harmful pores; however, at high replacement levels, the fraction of harmful and macropores may increase. Moreover, the fractal dimension is negatively correlated with the permeability grade and residual strength, but positively correlated with the strength degradation rate.

## 1. Introduction

Lightweight concrete has been increasingly adopted in civil engineering applications because it offers a rare balance of mechanical robustness, lightweight design, structural integrity, eco-conscious properties, and economic viability [1,2,3]. One of the fundamental differences between lightweight concrete and conventional cement-based concrete is the incorporation of lightweight aggregates, including materials like scoria [4], pumice stone [5], and particularly ceramsite [6]. Over the past few years, coal gangue ceramsite has seen widespread application in lightweight concrete thanks to its low thermal conductivity, excellent insulation properties, abundant availability, and well-established industrial production [7,8,9]. In contrast to conventional cement concrete, ceramsite lightweight concrete can reduce structural self-weight by over 20% without compromising strength [10,11], making it increasingly favoured in engineering projects.

Concrete permeability refers to the ability of the material to resist the ingress of pressurized water and is widely regarded as a critical indicator of long-term durability and service performance [12,13,14]. Ideal permeability performance plays a crucial role in limiting the ingress of moisture, aggressive ions, and other corrosive agents into concrete, which is essential for improving structural durability and extending service life. With the growing use of lightweight aggregate concrete in engineering practice, increasing attention has been devoted to investigating the permeability characteristics of lightweight concrete in recent years [15,16,17,18,19]. For example, Guneyisi et al. [20] and Fan [21] investigated the permeability performance of the CLAC, concluding that it exhibited superior permeability characteristics. Li et al. [22] employed an orthogonal experimental design to investigate how the content and particle size of ceramsite influence concrete permeability grades. Gao et al. [23] and Li [24] evaluated the permeability behavior of concrete prepared with different aggregate types. Their findings indicated that lightweight ceramsite exhibited the greatest permeability, followed by high-strength ceramsite, while crushed stone aggregates showed comparatively lower values. Kong et al. [25] examined the permeability behavior of concretes with varying water–binder ratios incorporating lightweight aggregates of different types and sizes, and reported that aggregate particle size plays a dominant role in governing concrete permeability.

However, most existing studies primarily focus on macroscopic permeability measurements, leaving the microscopic mechanisms linking pore structure to permeability largely unexplored. In particular, the combined influence of ceramsite particle size and replacement ratio on pore connectivity, pore structure evolution, and the resulting mechanical and permeability properties remains insufficiently investigated. Understanding these mechanisms is crucial not only for practical design optimization but also for advancing the theoretical knowledge of lightweight concrete microstructure–performance relationships.

Mercury intrusion porosimetry (MIP) is one of the most widely used techniques for characterizing the pore structure of cement-based materials, providing important information on pore size distribution, pore volume, and pore-related structural characteristics. Fractal theory can effectively characterize the complexity and heterogeneity of pore structures [26,27,28]. Compared with traditional pore structure parameters, fractal analysis can better reflect the geometric characteristics and connectivity of pore systems, which is beneficial for revealing the intrinsic relationship between pore structure evolution and permeability performance [29]. Using MIP combined with fractal theory, several researchers have quantitatively explored the relationship between pore structure characteristics and macroscopic concrete properties, such as compressive strength and permeability grades. For example, Aghililotf et al. [30], based on MIP analysis, found that higher porosity and interfacial microcracks weaken concrete strength while enhancing water penetration, whereas mineral admixtures can improve concrete performance by optimizing the pore structure. Rieg et al. [31] further confirmed through pore structure characterization that compressive strength is strongly correlated with porosity, pore volume, and pore tortuosity. Abounina et al. [32] pointed out that porosity, pore size distribution, and pore connectivity are key factors affecting the strength and permeability of porous concrete, among which total porosity has a particularly significant influence on compressive strength. Yavuz et al. [33] reported that an increase in porosity improves the water permeability of concrete but leads to a reduction in compressive strength. However, most of these studies focus on conventional concrete or single-size aggregates, and a systematic investigation of lightweight ceramsite concrete, considering both aggregate particle size and replacement ratio, is still lacking.

Hence, the present study systematically investigates the effects of ceramsite particle size and replacement ratio on the permeability and mechanical performance of lightweight concrete, employing MIP combined with fractal analysis to characterize pore structure and connectivity. By linking microstructural characteristics with macroscopic properties, this study provides both theoretical insights into the structure–property relationships of lightweight concrete and practical guidance for its optimized design in engineering applications.

## 2. Materials and Methods

### 2.1. Materials

**Figure 1:**
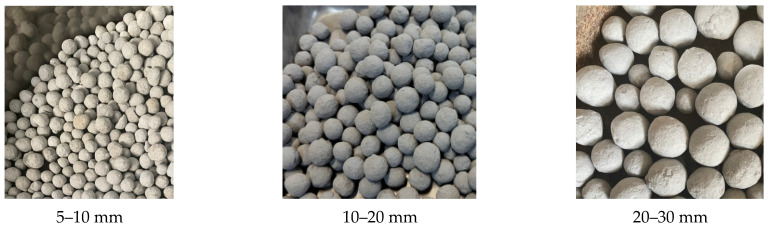
Coal gangue ceramsite.

**Figure 2:**
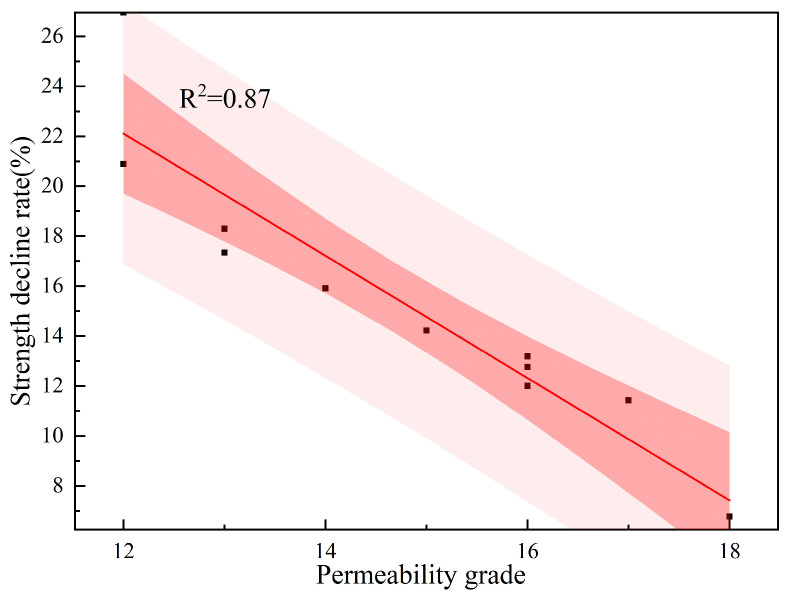
The relationship between permeability grade and strength decline rate.

CLAC mixtures were prepared with P.O 42.5 Portland cement, coal gangue ceramsite aggregates, natural aggregates, sand, coal gangue, silica fume, and a polycarboxylate-based water-reducing admixture. The ceramsites (see Figure 1 [22]) and basalt aggregates used in this study were supplied by Ningbo Zhongjin Environmental Protection Technology Co., Ltd. (Ningbo, China). Both types of aggregates were separated into three particle size ranges: 5–10 mm, 10–20 mm, and 20–30 mm. The grading curves of the three natural aggregates and the three ceramsites are shown in Figure 2. The coal gangue (grade II) and silica fume were provided from Hebei Boheng Minerals Co., Ltd. (Baoding, China). The polycarboxylate-based superplasticizer was obtained from Laiyang Hongxiang Building Admixture Co., Ltd. (Yantai, China).

### 2.2. Methods

#### 2.2.1. Specimen Preparation

The preparation of CLAC specimens strictly followed the GB/T 50080-2016 [34]. All raw materials were accurately weighed according to the experimental mix proportions. Before mixing, the coal gangue ceramsite aggregates were oven-dried at 105 ± 5 °C until constant mass and subsequently pre-soaked in water for 1 h. After pre-soaking, the moisture content of the ceramsite was measured and considered together with the designed water-to-binder ratio during specimen preparation. This pre-treatment was intended to reduce the influence of ceramsite water absorption on the effective mixing water and to improve the consistency of the mixtures.

The mixing process was conducted using an HJW-60 experimental concrete mixer (Cangzhou Lu Tong Wei Ye Testing Instrument Co., Ltd., Xian County, Cangzhou, China). The dry materials (cement, sand, aggregates, coal gangue, and silica fume) were first stirred for 60 s. Water and the superplasticizer were then added evenly, followed by an additional 120 s of wet mixing. To prevent ceramsite floatation and ensure structural density, the fresh concrete was cast into 150 mm × 175 mm × 185 mm molds in two layers. Each layer was consolidated using manual rodding and mechanical vibration on an HZJ-1 vibration table.

After leveling the surfaces, the specimens were covered with plastic sheeting to prevent moisture loss. After 24 h of curing at room temperature, the specimens were demolded using a vacuum demolding pump and transferred to a standard curing room for a 28-day period. For each group, twelve specimens were prepared: six for initial compressive strength tests and six for permeability and subsequent residual strength tests. Microstructural characterization, including MIP and SEM, was performed on samples extracted from non-permeated and post-permeability specimens, respectively.

#### 2.2.2. Laboratory Experiment

The compressive strength test and permeability test were conducted in accordance with the “Testing Methods of Cement and Concrete for Highway Engineering (JTG 3420-2020)” [35]. The axial compressive strength test of concrete prisms followed method T 0555-2005.

(1) Compression test

For the compressive strength test, a microcomputer-controlled electro-hydraulic servo universal testing machine was employed. Before testing, all specimens were subjected to standard curing for 28 days under a curing temperature of 20 ± 2 °C and relative humidity above 95%.

- After curing, the specimens were removed from the curing chamber and covered with a damp cloth to minimize moisture loss before testing.
- Prior to loading, the specimen surfaces were wiped dry and the dimensions were measured with an accuracy of 1 mm.
- The specimens were then centrally positioned on the lower platen of the testing machine to ensure geometric alignment.
- A loading rate of 0.5 MPa/s was applied continuously until failure. When rapid deformation occurred near the failure stage, the loading adjustment was stopped until the specimen was completely damaged, and the ultimate load was recorded.

The compressive strength was calculated using Equation (1). For each mixture, the final compressive strength value was determined as the average of three specimens, with the result reported to an accuracy of 0.1 MPa.

(1) $$f_{cp}=\frac{F}{A}$$

where *f_cp_* was the axial compressive strength of concrete prismatic body (MPa); *F* was the ultimate Load (N); *A* was the area under pressure (mm^2^).

(2) Permeability test

The permeability test was conducted using an HP-4.0 concrete digital display impermeability meter (Shanghai Qige Industrial Co., Ltd., Shanghai, China). The steps were as follows:

- After the test piece surface is thoroughly dried, apply rubber bands and sealant on both sides to ensure a good sealing performance. The sealant is prepared by mixing cement and butter in a ratio of 2:1 by weight. Additionally, two rubber bands are fixed at one-third and two-thirds of the height of the specimen respectively to further enhance the sealing effect.
- After sealing, the specimens were slowly pressed into the test mold using a hydraulic press until the bottom surface of the specimen was flush with the edge of the mold. The mold assembly was then installed onto the impermeability apparatus.
- Before installation, it is necessary to perform an exhaust treatment on the anti-seepage device to remove the internal air and avoid interfering with the test results.
- During testing, the initial water pressure was set to 0.1 MPa and automatically increased by 0.1 MPa every 8 h. The specimen surfaces were continuously monitored throughout the test. When water seepage appeared on the top surfaces of three out of six specimens, the corresponding water pressure was recorded and the test was terminated. If no seepage occurred after maintaining the designed pressure level for 8 h, the concrete was considered to satisfy the impermeability requirement.

The permeability grade was calculated according to Equation (2).

(2) P=10H−1

where *P* represents the permeability grade and *H* denotes the water pressure.

(3) Mercury pressure test (MIP)

The micro-pore structure of the specimens was investigated using a high-performance automatic mercury injection instrument (AutoPore V9620, Micromeritics Instruments Corporation, Norcross, GA, USA; supplied by Micromeritics Instrument (Shanghai) Co., Ltd., Shanghai, China). To ensure the reliability of the MIP results, the sample preparation followed a rigorous procedure: cubic fragments approximately 5–10 mm in size were extracted from the interior of the 28-day cured specimens. The hydration process was immediately terminated by immersing the samples in absolute ethanol. Prior to testing, the samples were dried in an oven at 80 °C until a constant weight was achieved to meet the high vacuum requirements of the MIP system. The mass of each tested sample was maintained at approximately 2–3 g to ensure representativeness. During the test, the mercury contact angle was set at 130°. Mercury was gradually intruded into the pores by increasing the external pressure up to a maximum of 227.50 MPa, allowing for the measurement of pore diameters as small as 5 nm.

(4) Scanning electron microscopy (SEM)

The microstructural characteristics of the interfacial transition zone (ITZ) between ceramsite and cement mortar, as well as between natural aggregate and cement mortar, were investigated using a high-resolution field emission scanning electron microscope (SEM, Carl Zeiss Microscopy GmbH, Oberkochen, Germany). The SEM system operated under high-vacuum conditions with a secondary electron (SE) imaging mode and an ultra-low voltage resolution of 0.9 nm.

Prior to SEM observation, the cement-based specimens were dried and prepared as thin flaky samples. The specimen stage was first cleaned using absorbent cotton soaked with alcohol and dried with compressed air. Conductive adhesive tape was attached to the specimen stage to improve electrical conductivity. The dried specimens were then fixed onto the conductive tape, and additional conductive strips were applied to ensure stable conductivity during testing. Subsequently, the samples were vacuum-treated and gold-coated before SEM observation. The prepared specimens were finally placed into the SEM chamber for microstructural analysis, and images were collected at magnifications of 1000×.

## 3. Experimental Investigation for Permeability Properties

To more clearly investigate the influence of ceramsite particle size on the permeability and pore structure characteristics of CLAC, only one ceramsite particle size was incorporated in each mixture group. This experimental design was intended to minimize the interaction among different particle sizes and to facilitate comparative analysis of the influence of ceramsite size.

In addition, based on preliminary experimental observations, the incorporation of 5–10 mm ceramsite showed relatively favorable effects on the mechanical and permeability-related properties of concrete, whereas excessive incorporation of 10–20 mm and 20–30 mm ceramsite tended to negatively affect the concrete performance. Therefore, a relatively larger number of replacement levels and experimental groups were designed for the 5–10 mm ceramsite mixtures in order to more thoroughly investigate its influence on concrete properties. The selection of the content was also based on existing research [36,37]. The ceramsite replacement ratio adopted in this study was based on equal-volume replacement of natural coarse aggregate, as shown in Equation (3).

(3) $$X=\frac{m_{2}\rho_{1}}{m_{1}\rho_{2}}$$

where *m*_1_ is the mass of the natural aggregate before replacement; *ρ*_1_ is the density of the natural aggregate; *m*_2_ is the mass of the ceramsite; and *ρ*_2_ is the density of the ceramsite.

To gain a clearer understanding of the permeability properties of the CLAC, its mix proportions were designed, and tests were conducted on compressive strength, permeability grade, and residual strength. This study examined the mechanical performance and permeability of concrete, with the specific combinations used listed in Table 1.

The compressive strength, permeability grade, residual strength and strength decline rate are shown in Table 2.

As shown in Table 2, compared with ordinary concrete, CLAC exhibits a marginal reduction in compressive strength, indicating slightly inferior mechanical performance. In contrast, CLAC demonstrates a higher permeability grade, reflecting its enhanced permeability characteristics relative to conventional concrete. In this study, CRR1, CRR2, and CRR3 represent 5–10 mm, 10–20 mm, and 20–30 mm ceramsite replacement ratio, respectively.

As the contents of CRR3 and CRR2 increase, the compressive strength, permeability grade, and residual strength of CLAC all decrease simultaneously. This indicates that the incorporation of 20–30 mm and 10–20 mm ceramsite has an adverse effect on both the mechanical performance and permeability characteristics of CLAC. Moreover, as CRR3 increases, the strength decline rate of CLAC increases, indicating that adding too much 20–30 mm ceramsite is also detrimental to the mechanical properties of CLAC. In contrast, as CRR1 increases, the compressive strength, permeability grade, and residual strength of CLAC all increase, while the strength decline rate decreases, indicating that the addition of 5–10 mm ceramsite has a beneficial effect on both the mechanical performance and permeability characteristics of CLAC. Furthermore, a negative correlation exists between permeability grade and strength degradation rate: higher permeability grades correspond to lower strength degradation rates, and vice versa. This indicates that when CLAC exhibits satisfactory permeability, its mechanical properties are correspondingly favorable.

## 4. Experimental Investigation of Pore Structure

### 4.1. Distribution of Pore Size

Different pore classification systems have been widely adopted in cement-based material research for different analytical purposes. The harmful pore classification system mainly reflects the potential influence of pores on concrete durability and mechanical performance, whereas the traditional pore size classification system (gel pores, transition pores, capillary pores, and macropores) is commonly used to analyze pore size distribution and transport-related characteristics. In this study, the selection of these two pore classification methods is based on the research conducted by Zhang et al. [38] on the characterization of pore structures in cement-based materials. Therefore, the trends obtained from the two systems are not expected to be identical and should be interpreted within the context of each classification criterion.

To enhance the pore size analysis, the classification approach proposed by Wu [39] was adopted, dividing the pore sizes into four categories: harmless pores (<20 nm), less harmful pores (20–100 nm), harmful pores (100–200 nm), and more harmful pores (>200 nm). This classification method can more intuitively reflect the distribution characteristics of pores at different scales within concrete and is helpful for analyzing the variation in pore structure after the incorporation of ceramsite. Based on this classification system, the proportions of different pore types in CLAC were calculated, as presented in Table 3. Through this classification approach, the variation trends of pore size distribution under different ceramsite particle sizes and replacement ratios can be more clearly compared, thereby providing a better understanding of the influence of ceramsite on the pore structure evolution of concrete.

**Figure 3:**
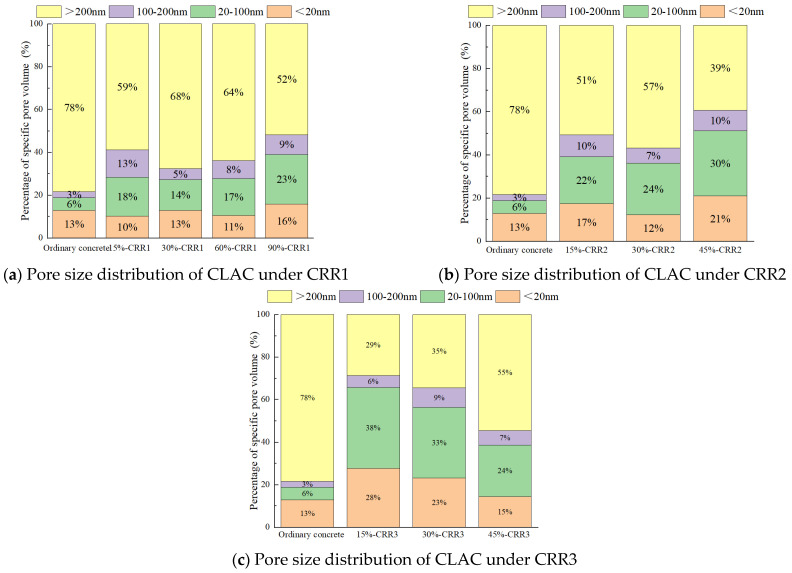
Pore volume percentage according to harmful pore classification.

According to the pore size classification method proposed by Wu [40], the pore volume percentage of the CLAC is shown in Figure 3.

As shown in Figure 3, the incorporation of ceramsite with different particle sizes leads to a noticeable refinement of the pore structure in CLAC. Compared with ordinary concrete, CLAC at moderate ceramsite content exhibits a higher fraction of harmless pores and a relative decrease in harmful pores; however, with further increase in ceramsite replacement ratio (especially CRR2 and CRR3), the fraction of harmful and macropores increases. Consequently, CLAC presents a more favorable pore system than conventional concrete, which is conducive to improved overall performance.

As shown in Figure 3a, variations in CRR1 markedly influence the pore composition of CLAC. With increasing CRR1, the contents of harmless and less harmful pores generally increase, whereas the fraction of harmful pores exhibits a rise followed by a decline. Specifically, as CRR1 increases from 15% to 90%, the share of harmless pores increases from approximately 10% to 16%, while less harmful pores rise from about 18% to 23%. However, at CRR1 levels of 30% and 60%, the proportion of less harmful pores decreases noticeably, accounting for only 14% and 17%, respectively. In addition, the content of harmless pores increases from 59% to 68% when CRR1 ranges from 10% to 30%, but subsequently declines from 68% to 52% as CRR1 further increases from 30% to 90%. The reductions observed at CRR1 values of 30% and 60% suggest the presence of a critical threshold affecting the distribution of less harmful pores. Beyond this point, the increase in ceramsite may instead have a negative impact on the formation of fine pores. This phenomenon may be attributed to the interaction between ceramsite and the relatively limited cement paste. Adding ceramsite alters pore connectivity, creating a more intricate pore network. At moderate replacement levels, this leads to the formation of more harmless and less harmful pores. However, excessive ceramsite replacement increases the structural complexity, which can reduce the fraction of harmless pores. Initially, a rise in CRR1 results in a higher proportion of harmful pores, likely due to the incomplete bonding between ceramsite and cement paste, generating larger voids. Beyond a certain replacement threshold, further addition of ceramsite enhances overall pore compactness, thereby decreasing the proportion of harmful pores.

As shown in Figure 3b, increasing CRR2 leads to higher fractions of harmless pores and those with harmful pores, whereas the fraction of harmful pores initially rises and then declines. Specifically, as CRR2 increases from 15% to 45%, the proportion of harmless pores grows from 17% to 21%, and pores with harmful increase from 22% to 30%, while highly harmful pores rise from 51% to 57%. Beyond this range, further increases in CRR2 result in a reduction in the fraction of harmful pores, dropping from 57% to 39%. This phenomenon can be attributed to the moderate particle size of 10–20 mm ceramsite, which effectively fills concrete pores and promotes the formation of smaller, less detrimental voids. When combined with cement paste, this particle size encourages the development of finer pores, ultimately enhancing the fraction of harmless voids. As CRR2 increases, the optimization of concrete microstructure results in a higher proportion of both harmless and harmful pores. Initially, however, the proportion of harmful pores increases due to the larger ceramsite particles, which may lead to pore redistribution and an increase in the number of harmful voids. However, further increasing CRR2 strengthens the bond between ceramsite and cement paste, which can fill or stabilize larger voids, resulting in a reduction in harmful pores. This observation highlights the balancing effect within the internal pore network of ceramsite-based lightweight aggregate concrete. At a given replacement level, interactions among harmless, slightly harmful, and highly harmful pores influence the overall pore distribution. As the ceramsite replacement ratio, the system undergoes a readjustment, ultimately optimizing the microstructural arrangement of pores.

As shown in Figure 3c, increasing CRR3 leads to a decline in the fraction of harmless pores, while the fraction of harmful pores rises progressively. Specifically, when CRR3 increases from 15% to 45%, harmless pores decrease from 28% to 15%, whereas harmful pores grow from 29% to 55%. This might be due to the fact that 20–30 mm ceramsite occupies a relatively large space in the concrete, which could lead to a change in the overall pore structure. As CRR3 increases, the likelihood of harmless pores being compressed or filled diminishes, resulting in a reduced fraction of these pores. Furthermore, the addition of 20–30 mm ceramsite can hinder the cement paste from effectively filling finer voids, leading to a simultaneous decrease in harmless pores and an increase in harmful pores. This limited filling capacity influences the overall pore network. Additionally, the incorporation of 20–30 mm ceramsite may cause pore redistribution, further promoting the formation of harmful voids. This is because larger gaps in the ceramsite may form larger pores, thereby increasing the proportion of harmful pores. Therefore, adding too much 20–30 mm particle size ceramsite may also cause the internal pore structure of the CLAC to deteriorate.

**Figure 4:**
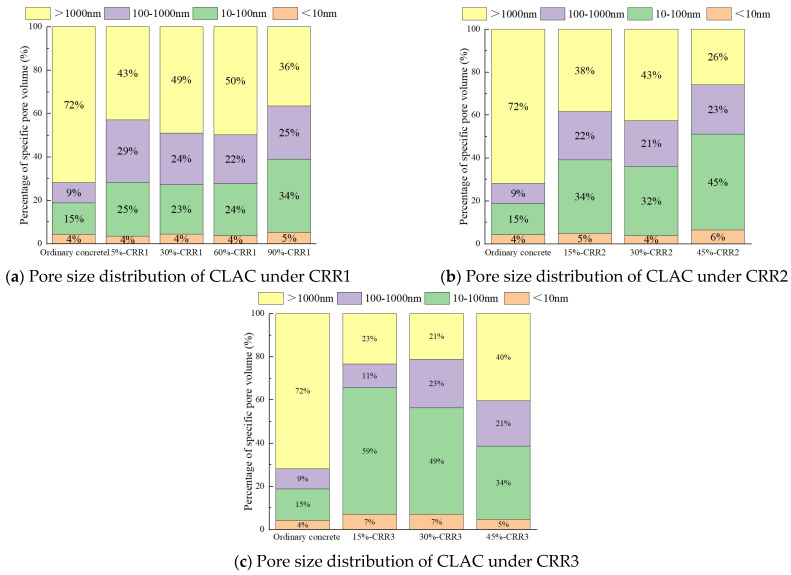
Percentage of specific pore volume.

Furthermore, to examine the effect of aggregates, the pore structure of CLAC was categorized into four classes based on pore size: gel pores (<10 nm), transition pores (10–100 nm), capillary pores (100–1000 nm), and macroscopic pores (>1000 nm) [41]. This classification system is mainly based on the functional role of pores in cement-based materials and is commonly used to analyze moisture transport, gas diffusion, and mechanical behavior. Specifically, gel pores are closely related to cement hydration products and mainly affect the strength and durability of concrete. Transition pores and capillary pores are generally associated with the transport properties of concrete, while macropores are more closely related to structural stability and mechanical deterioration behavior. Therefore, the two pore classification systems adopted in this study serve different analytical purposes and provide complementary information for understanding the pore structure evolution characteristics of CLAC. The proportion of the calculated aperture distribution is shown in Table 4. The percentage of pore volume is shown in Figure 4.

As shown in Figure 4a, incorporating 5–10 mm ceramsite increases the fractions of gel, transition, and capillary pores in CLAC, while reducing macroscopic pores. The combined fraction of gel and transition pores rises from 19% in ordinary concrete to 39%, whereas macroscopic pores decrease from 72% to 36%. Gel pore content remains relatively stable with increasing CRR1, showing a slight rise from 4% to 5% only at a 90% replacement level. Transition and capillary pores initially decline and then increase as CRR1 rises: transition pores drop from 25% to 23% when CRR1 increases from 15% to 30%, then recover to 34% at 90% CRR1; similarly, capillary pores decrease from 29% to 22% between 15–60% CRR1, and increase to 25% at 90%. Macroscopic pores exhibit a first increase followed by a decrease, rising from 43% to 50% for CRR1 values of 15–60%, and then declining to 36% as CRR1 reaches 90%.

As shown in Figure 4b, incorporating 10–20 mm ceramsite markedly increases the fractions of gel, transition, and capillary pores in CLAC, accompanied by a pronounced reduction in macroscopic pores. Compared with ordinary concrete, the combined fraction of gel and transition pores rises from 19% to 51%, whereas macroscopic pores decrease substantially from 72% to 26%. As CRR2 increases, gel, transition, and capillary pores exhibit a non-monotonic trend, initially declining and then increasing. Specifically, when CRR2 changes from 15% to 30%, gel pores decrease from 5% to 4%, transition pores from 34% to 32%, and capillary pores from 22% to 21%. With further increase in CRR2 from 30% to 45%, these pore fractions rebound, with gel pores increasing to 6%, transition pores to 45%, and capillary pores to 23%. In contrast, macroscopic pores display an opposite evolution, first rising from 38% to 43% at CRR2 levels of 15–30%, and subsequently declining to 26% as CRR2 reaches 45%.

As shown in Figure 4c, incorporating 20–30 mm ceramsite increases the fractions of gel, transition, and capillary pores in CLAC, while reducing macroscopic pores. The combined fraction of gel and transition pores rises from 19% in ordinary concrete to 66%, whereas macroscopic pores decrease from 72% to 21%. However, as CRR3 increases, the contents of gel and transition pores continuously decrease, while the proportion of macroscopic pores continuously increases. Specifically, when CRR3 increases from 15% to 45%, the fraction of gel pores reduces from 7% to 5%, and transition pores decrease from 59% to 34%. Capillary pores show a non-monotonic trend, initially increasing from 11% to 23% between 15% and 30% CRR3, then decreasing from 23% to 21% as CRR3 rises from 30% to 45%. Similarly, macroscopic pores first decrease slightly from 23% to 21% as CRR3 increases from 15% to 30%, and then increase sharply from 21% to 40% as CRR3 increases from 30% to 45%, indicating that an increase in the replacement ratio deteriorates the pore structure of CLAC.

Furthermore, regarding the situation where the proportion of macroscopic pores is relatively high, there are certain limitations when applying the mercury intrusion porosimetry method in lightweight aggregate concrete systems. Unlike conventional dense concrete, coal gangue ceramsite contains a highly porous internal structure and interconnected open pores, which can significantly contribute to mercury intrusion in the larger pore diameter range. Therefore, the reported macropore fraction reflects not only matrix pores, but also accessible pores associated with the lightweight aggregate itself and the interfacial transition zones.

In addition, MIP fundamentally characterizes accessible pore throats rather than true pore body sizes, and the ink-bottle effect may influence the apparent pore size distribution. The pore structure results in this study are intended primarily for comparative analysis among mixtures rather than as absolute quantification of the intrinsic pore geometry.

By comparing (a–c) in Figure 3 and Figure 4, it is found that although in the ceramsite-containing CLAC groups, the proportions of harmful pores and macropores increase with the increase in ceramsite replacement ratio, compared with ordinary concrete, all three particle sizes of ceramsite can effectively refine the internal pores of CLAC. Among them, the 20–30 mm ceramsite exhibits the most significant refining effect, while the 5–10 mm ceramsite shows the least obvious refining effect.

It’s worth noting that in lightweight aggregate concrete systems, the macropore fraction measured by MIP includes pores inside the ceramsite aggregates and the ITZ, and does not fully represent the matrix pores. MIP inherently characterizes accessible pore throats, and the ink-bottle effect may cause measurement deviations for small pores. Therefore, the MIP data in this study are primarily used for comparing relative trends among different mix designs rather than absolute pore sizes or pore volumes [42].

### 4.2. Pore Structure Parameters

**Figure 5:**
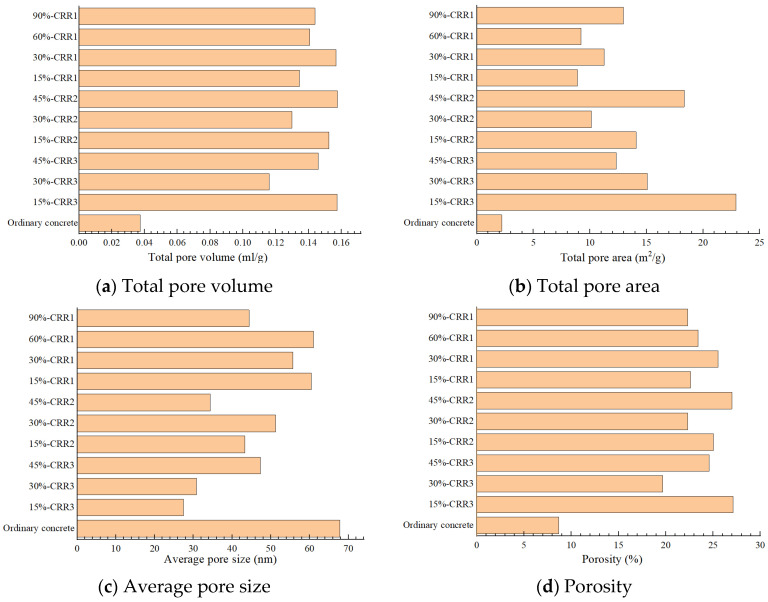
Pore structure parameter.

The pore structure parameters of ordinary concrete and lightweight concrete are shown in Figure 5.

As shown in Figure 5, the incorporation of ceramsite leads to pronounced changes in the pore characteristics of CLAC. With increasing ceramsite content, both porosity and the associated total pore volume and total pore area exhibit an overall upward tendency, whereas the average pore size shows a decreasing trend. This behavior suggests that ceramsite contributes to pore refinement by redistributing the pore system toward finer scales. The observed increase in porosity can be attributed to modifications at the interfacial transition zone induced by ceramsite incorporation. Furthermore, variations in ceramsite particle size result in distinct pore evolution patterns as the replacement level changes. Owing to its relatively uniform morphology and rough surface texture, ceramsite improves aggregate packing efficiency, limits the development of large pores, and promotes the formation of smaller pores. Consequently, the pore system in CLAC is characterized by fewer large-diameter pores and a higher proportion of fine pores. In addition, enhanced bonding between the cement matrix and aggregates further stabilizes the internal structure, contributing to a reduction in pore size and connectivity.

## 5. Fractal Approach for Pore Structure

According to existing studies, the pore structure of cement-based materials exhibits obvious fractal characteristics. Based on MIP, several fractal models have been developed to characterize pore structures, mainly including the space-filling model, the Menger sponge model, the pore-axis fractal model, and thermodynamic fractal models. The space-filling model describes the spatial distribution of pores in three-dimensional space, while the pore-axis fractal model focuses on the geometrical characteristics and distribution features of pores. Thermodynamic fractal models are mainly used to investigate the relationship between pore structure and thermodynamic properties. Among these models, the Menger sponge model is the most widely adopted and considers pores and cracks in concrete as a sponge-like porous structure. Different fractal models may yield different fractal dimension results. Recent studies have suggested that fractal dimensions can serve as complementary parameters for interpreting transport-related properties of cementitious materials [43].

Many researchers have applied the Menger sponge model to characterize the fractal characteristics of concrete pore systems based on MIP data [44]. The model provides a useful comparative description of the irregularity and spatial heterogeneity of the accessible pore network. In topology, the Menger sponge has also been used to simulate irregular objects such as tree branches, road networks, and coastlines. The fractal theory of the Menger sponge describes irregular fractal objects through infinite iterations of a fixed mathematical model. Therefore, the Menger sponge model has been widely used to comparatively characterize the geometric complexity and heterogeneity of pore systems in cement-based materials [45]. In this model, the fractal dimension is used to characterize the complexity of the internal pore system in concrete [46,47].

In this study, the Menger sponge model was employed to describe the internal pore characteristics of concrete [48,49,50]. The model construction procedure followed the approach proposed by Lin et al. [51].

### 5.1. Fractal Dimension

**Figure 6:**
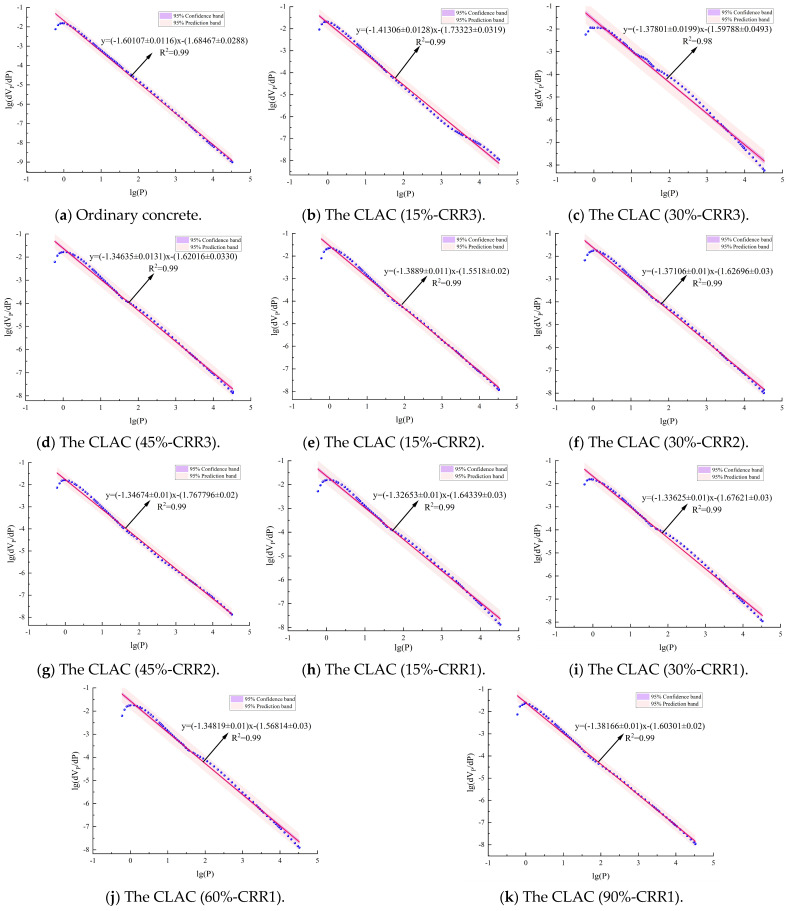
Pore surface fractal dimension.

The surface fractal dimension (SFD) results of ordinary concrete and the CLAC are shown in Figure 6. The fractal dimension of the pore surface is shown in Table 5.

According to fractal theory, the SFD of porous objects usually ranges from 2 to 3. When D approaches the value of 3, it indicates that the pore system exhibits increased geometric complexity, accompanied by a highly heterogeneous spatial arrangement. On the contrary, when D approaches the value of 2, the pore structure tends to be smooth, approaching a flat surface. As shown in Figure 6, the SFD of the CLAC is 2.5869–2.6735, which is significantly higher than that of ordinary concrete, which is 2.3989. This result indicates that with the introduction of ceramsite, the surface irregularity of internal pores and cracks in concrete has been significantly enhanced. The SFD is an indicator for measuring the complexity of an object. A higher SFD indicates increased structural heterogeneity and greater irregularity in pore geometry.

In the CLAC, the addition of ceramsite promotes the diversification and complication of pore morphology, which may be closely related to the particle characteristics of ceramsite itself and its distribution pattern in concrete. This irregular pore configuration contributes to improved durability performance by restricting the ingress of water and other aggressive agents. Furthermore, the correlation coefficients in the table are all greater than 0.985. These statistical results indicate that the model provides relatively consistent fitting behavior for the accessible pore structure data obtained from MIP. This means that the model can effectively capture the subtle differences in pore structure between the two types of concrete, and can more effectively reflect the fractal characteristics of the internal pores of ordinary concrete and the CLAC.

For the larger ceramsite particles (10–20 mm and 20–30 mm), increasing replacement ratio leads to a more open skeleton and complex pore network, enhancing the fractal dimension due to larger and more irregular voids and ITZs. In contrast, for the smaller 5–10 mm particles, increasing replacement ratio promotes a filling effect, densifying the aggregate skeleton, reducing pore network complexity, and thus decreasing the fractal dimension.

**Figure 7:**
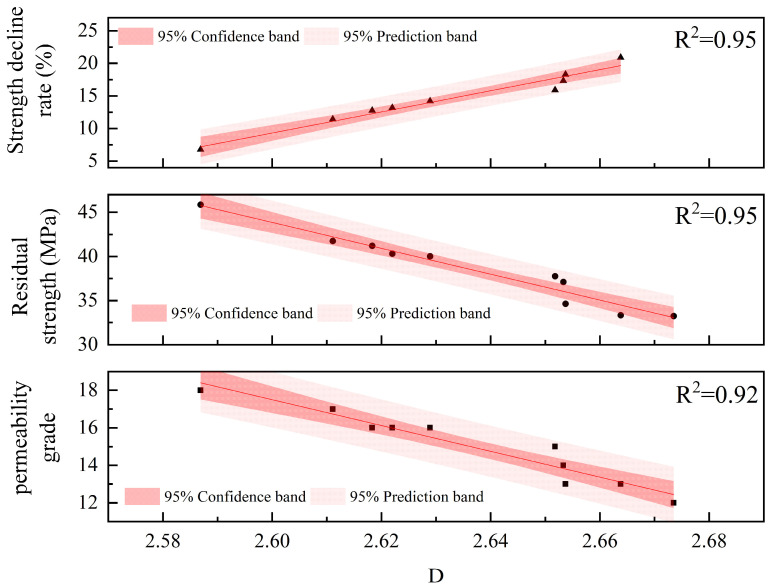
Relationship between the SFD and permeability grade, residual strength, and strength decline rate.

The relationship fitting between the SFD and the permeability grade, residual strength, and strength decline rate is shown in Figure 7.

As shown in Figure 7, there are corresponding relationships between the SFD and the permeability grade, residual strength, and strength decline rate. A negative correlation between the SFD and the permeability grade can be observed. This may be related to the changes in pore structure characteristics and pore distribution caused by ceramsite incorporation. The increase in SFD reflects the increased geometrical irregularity and distribution complexity of the pore structure in ceramsite lightweight aggregate concrete, which may lead to a higher proportion of interconnected harmful pores and thus reduce the permeability resistance. The results also indicate a linear association between SFD and residual strength, with a correlation coefficient of 0.95 (in absolute value). As the SFD increases, the residual strength of CLAC exhibits a downward tendency. This behavior may be associated with the coarsening of pore structure and the increase in harmful pores in the concrete system, rather than the increase in fractal dimension itself. In addition, SFD shows a positive relationship with the strength decline rate. With increasing SFD, the strength decline rate of CLAC increases.

By comparison, it is found that the absolute fitting coefficient between the SFD and the strength decline rate is the largest (R^2^ = 0.95), that is, the correlation between the SFD and the strength decline rate is the highest, which is reasonable. SFD serves as an indicator describing the complexity and spatial arrangement of pores at the microscale within concrete. The strength degradation behavior is primarily governed by the characteristics of the internal pore network and microstructural features, especially the presence of harmful pores and microcracks. Therefore, the change in the SFD has a more direct impact on the strength decline rate. However, permeability properties such as permeability grade are not only affected by the internal microstructure of concrete, but also influenced by multiple factors including the overall density of concrete, material composition, and proportion. Therefore, the correlation between the SFD and the strength decline rate is higher than that between the SFD and the permeability grade and the residual strength.

### 5.2. Analysis of Fractal Dimension Characteristics

To obtain a more detailed and reliable description of the concrete pore system, the fractal dimensions of pore surfaces corresponding to different pore-size ranges were determined. Based on pore diameter, the pores were categorized into four distinct regions:

- Region One: macroscopic pores (>1000 nm),
- Region Two: capillary pores (100–1000 nm),
- Region three: transition holes (10–100 nm),
- Region Four: gel pores (<10 nm).

The computational fractal dimensions corresponding to the four regions are D_m_, D_c_, D_t_, and D_g_. The results of SFD for each region are shown in Table 5.

As shown in Table 5, it can be found that the SFD of region one is significantly greater than that of the other three regions. This indicates that a greater pore size corresponds to a higher level of complexity in the pores. This is because macroscopic pores usually refer to larger-sized pores, whose shapes are often more complex and can form diverse geometric shapes and structures. This complexity is reflected in the higher D. In contrast, the size of capillary pores, transition pores and gel pores are smaller, and their morphology and structure are relatively simple, resulting in a lower D. On the other hand, larger pores usually have more interconnections and intersections, forming a more complex pore network. This interconnectivity increases the overall complexity of the pore structure, thereby enhancing the SFD. However, due to the size limitations of small holes, the way they are connected to each other is relatively simple, resulting in a reduction in the SFD.

The fractal dimensions of Region One, Region Two, and Region Three in ordinary concrete are noticeably lower than those observed in ceramsite lightweight aggregate concrete. Yu et al. [52] reported a high prevalence of ink bottle pores with sizes below 100 nm, while Zhang et al. [53] confirmed that the ink bottle effect also occurs in capillary and gel pores. The expanded interfacial area between ceramsite and the surrounding concrete contributes to the formation of additional capillary and transition pores, resulting in higher fractal dimensions in all three regions. The differences in the fractal dimension Dg among regions four are not as obvious as those between Regions One Dm and Two Dc. This result can be attributed to the fact that the formation of gel pores mainly depends on hydration reactions, and the changes in their morphology and size among different materials are relatively small. Hence, the differences in SFD are also limited.

It should be noted that the MIP-based fractal dimensions obtained in this study are influenced by the inherent limitations of mercury intrusion porosimetry, including pore-throat measurement characteristics, ink-bottle effects, and possible mercury intrusion into accessible ceramsite pores. Therefore, the calculated fractal dimensions are mainly intended for comparative analysis among different mix designs rather than absolute quantification of the intrinsic pore geometry.

### 5.3. The Interrelationship Among Pore Parameters

**Figure 8:**
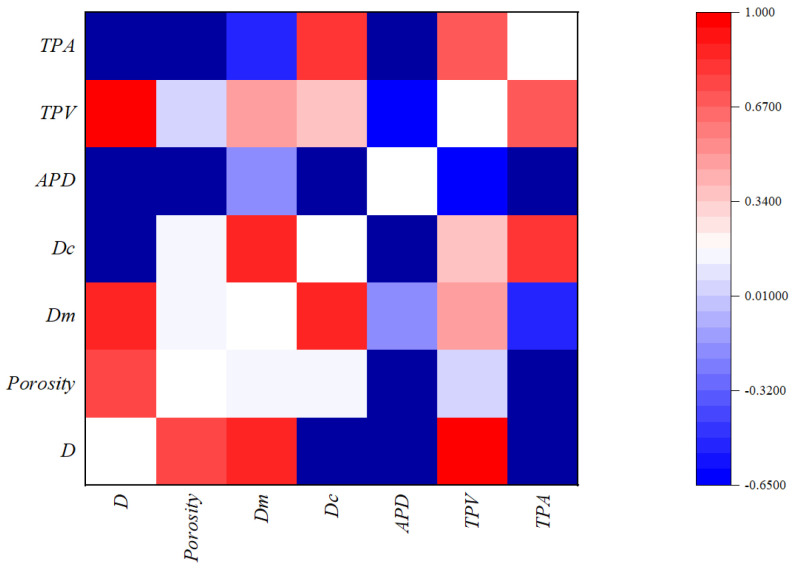
Correlation analysis results among pore parameters.

To establish the relationships among various pore parameters, correlation analysis was performed using the Pearson correlation coefficient on SFD, porosity, Dm, Dc, average pore size (APD), total pore volume (TPV), and total pore area (TPA). The result is shown in Figure 8.

As shown in Figure 8, SFD exhibits little correlation with porosity, suggesting that porosity alone cannot adequately capture the intricacies of the pore structure. The correlation between the SFD and the total pore volume, Dm, and average pore diameter is significant, and the correlation coefficient between the SFD and the total pore volume is the highest. This is because complex pore structures usually contain more branches and fine structures, which leads to a corresponding increase in the total volume of pores and the number of pores. A larger pore volume usually indicates the presence of more pores and a more complex pore structure. When the pore volume of a material increases, the SFD often increases as well, because more pores and more complex structures lead to higher self-similarity. As the SFD increases, the average pore size decreases, indicating that the smaller the pore size, the higher the complexity of the pore structure.

In addition, it can also be observed that gel pores exhibit a good correlation with capillary pores. This is because the formation of the two types of pores is usually influenced by similar physical and chemical mechanisms. For instance, during the gelation process, the flow of the liquid and the formation of bubbles may lead to capillary phenomena, thereby generating capillary pores. The properties of the gel (such as viscosity, surface tension, etc.) will affect its pore structure, and the same factors will also play a role in the formation of capillary pores. This similarity makes them show a good correlation in statistical analysis.

## 6. Micro-Structure

**Figure 9:**
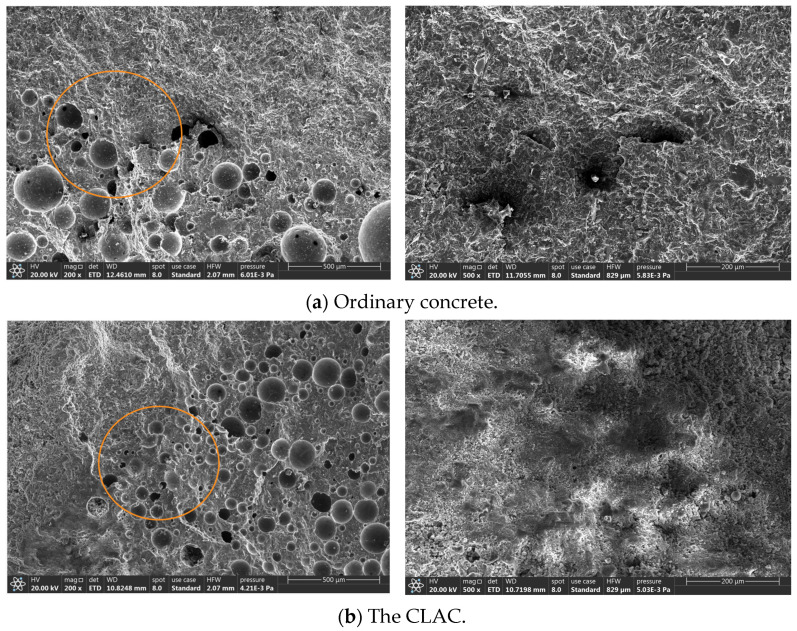
Concrete interface transition zone.

Recent studies have shown that the interfacial transition zone (ITZ) in lightweight aggregate concrete plays an important role in transport and mechanical behavior, and may differ significantly from that of normal-weight concrete [54]. To further explore the permeability characteristics of the CLAC, Figure 9 presents the microscopic morphology inside the CLAC through SEM experiments.

As shown in Figure 9, it can be seen that there are differences in the microstructure morphology of the aggregate—mortar interface transition zone between ordinary concrete and the CLAC. The interior of the ceramsite lightweight aggregate is a porous and irregular honeycomb-like structure. These randomly distributed pores give it characteristics such as low bulk density and low water absorption rate. During the hydration process, some cement particles will enter the pores of the ceramsite and undergo hydration reactions, which makes the bond between the cement stone and the ceramsite more complete. Therefore, it can be observed from the right image of Figure 9b that the boundary between the ceramsite aggregate and the cement matrix appears relatively indistinct in the observed region, suggesting relatively close contact between the two phases. The SEM observations qualitatively indicate a relatively compact interface transition zone between the ceramsite aggregate and cement mortar, without obvious large voids in the observed regions. The excellent interface bonding property forms a denser interface transition zone, thus improving the permeability of the CLAC.

**Figure 10:**
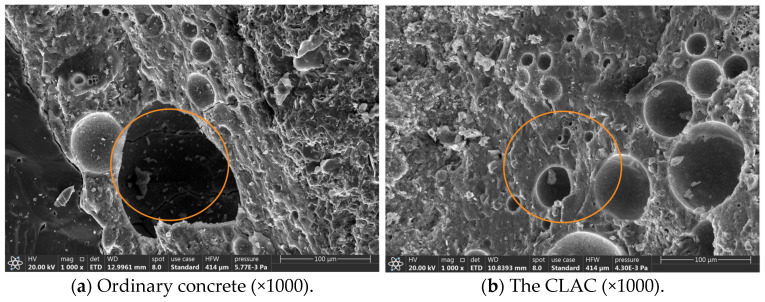
Internal pore structure.

As shown in Figure 10, when magnified 1000 times, the SEM images suggest that the pores inside the ceramsite aggregate appear relatively isolated in the observed regions. In contrast, the pores inside the ordinary aggregate have larger diameters and are interconnected. Moreover, in the ordinary aggregate, relatively obvious cracks run through the pores, serving as connecting bridges for capillary pores. Under low water pressure, water can easily pass through these cracks. Therefore, the permeability resistance of ordinary concrete is inferior to that of CLAC. Further combining with the SFD data in Section 5.1, it is found that the SFD of ordinary concrete is generally lower than that of CLAC. This microstructural feature may be associated with the observed differences in fractal dimension between ordinary concrete and CLAC. As shown in Figure 10, the observed pore morphology in CLAC appears less interconnected in the local SEM regions compared with ordinary concrete. Since SFD is closely related to pore connectivity and the complexity of permeation paths, this results in the lower fractal dimension of ordinary concrete compared to CLAC.

## 7. Conclusions

In this study, the influence of ceramsite size and replacement ratio on permeability characteristics of lightweight concrete was analyzed through a fractal approach. The following conclusions were obtained:

(1) The incorporation of ceramsite changed the accessible pore structure of CLAC, as characterized by MIP. At moderate ceramsite replacement levels, the fraction of harmful pores may decrease while harmless and less harmful pores increase; however, excessive ceramsite replacement can lead to an increase in harmful and macropores. Therefore, the pore structure evolution depends strongly on ceramsite particle size and replacement ratio. With increasing ceramsite content, total pore volume, total pore area, and overall porosity rise, while the average pore diameter shows a decreasing tendency. This further demonstrates that ceramsite contributes to the refinement of concrete internal pore structure.
(2) The introduction of ceramsite has significantly enhanced the surface irregularity of internal pores and cracks in concrete. The surface fractal dimension of CLAC increases with increasing CRR2 and CRR3, but decreases with increasing CRR1.
(3) The surface fractal dimension is negatively correlated with the permeability grade and residual strength, and positively correlated with the strength decay rate. The correlation coefficient between the surface fractal dimension and the permeability grade is 0.92, and the correlation coefficients with residual strength and strength decay rate are both 0.95, indicating that the surface fractal dimension exhibits strong correlations with all performance parameters.
(4) There are differences in the microscopic morphology of the aggregate–mortar interface transition zone between ordinary concrete and the ceramsite aggregate lightweight aggregate concrete. The interior of ceramsite aggregate lightweight aggregate has a porous characteristic structure, mainly small pores, which are not interconnected with each other. In contrast, the internal pore diameter of ordinary aggregate is larger and there is a situation of interconnection.
(5) The fractal dimension used in this study not only characterizes pore size variation, but also comprehensively reflects the complexity, tortuosity, and spatial heterogeneity of the pore network. Compared with traditional pore structure parameters such as porosity and average pore diameter, fractal analysis provides a more effective approach for linking microscopic pore characteristics with macroscopic durability-related properties.
(6) Among the tested mixtures, appropriate ceramsite replacement showed a favorable trend in permeability grade and residual strength. However, since only permeability grade was measured in this study, the results should be interpreted as permeability-related performance rather than comprehensive durability improvement.

In future study, the unique microstructure characteristics of coal gangue ceramsite will be combined to make a finer classification to determine the key particle size that affects the change in fractal dimension behavior. Moreover, freeze–thaw cycles, sulfate erosion, and carbonation will be conducted to compare the reliability of the fractal dimension as a prediction indicator. Finally, the permeability coefficient and the limitations associated with MIP, including ink-bottle effects and pore-throat accessibility, will be further investigated in future studies.

## Figures and Tables

**Table 1:** Design of mix proportion.

No.	Water–Binder Ratio	Fly Ash	Silica Fume	Ceramsite Replacement Ratio		
				5–10 mm	10–20 mm	20–30 mm
1	0.28	15%	10%	0%	0%	0%
2	0.28	15%	10%	0%	0%	15%
3	0.28	15%	10%	0%	0%	30%
4	0.28	15%	10%	0%	0%	45%
5	0.28	15%	10%	0%	15%	0%
6	0.28	15%	10%	0%	30%	0%
7	0.28	15%	10%	0%	45%	0%
8	0.28	15%	10%	15%	0%	0%
9	0.28	15%	10%	30%	0%	0%
10	0.28	15%	10%	60%	0%	0%
11	0.28	15%	10%	90%	0%	0%

**Table 2:** Test results.

No.	Compressive Strength (MPa)	Permeability Grade	Residual Strength (MPa)	Strength Decline Rate (%)
1	47.50	12	34.70	26.95
2	47.48	16	41.22	13.19
3	44.14	14	37.12	15.91
4	42.03	12	33.25	20.89
5	46.16	16	40.32	12.64
6	44.02	15	37.76	14.22
7	42.40	13	34.64	18.30
8	40.33	13	33.34	17.34
9	45.89	16	40.03	12.76
10	47.10	17	41.76	11.43
11	49.21	18	45.87	6.78

**Table 3:** Percentage of harmless, less harmful, harmful, and more harmful pores in CLAC.

No.	<20 nm	20–100 nm	100–200 nm	>200 nm
1	12.86%	5.98%	2.80%	78.36%
2	27.80%	37.88%	5.65%	28.68%
3	23.24%	33.01%	9.25%	34.50%
4	14.50%	24.13%	6.85%	54.52%
5	17.39%	21.80%	10.13%	50.67%
6	12.23%	23.88%	7.10%	56.79%
7	20.96%	30.16%	9.53%	39.34%
8	10.25%	18.03%	12.87%	58.85%
9	12.86%	14.34%	5.28%	67.53%
10	10.52%	17.27%	8.42%	63.80%
11	15.84%	23.17%	9.23%	51.76%

**Table 4:** Pore diameter distribution proportion of the CLAC.

No.	<10 nm	10–100 nm	100–1000 nm	>1000 nm
1	4.28%	14.56%	9.31%	71.85%
2	7.11%	58.57%	10.96%	23.36%
3	7.14%	49.11%	22.66%	21.09%
4	4.75%	33.88%	21.00%	40.37%
5	4.91%	34.28%	22.38%	38.42%
6	3.83%	32.29%	21.30%	42.58%
7	6.41%	44.72%	23.00%	25.86%
8	3.57%	24.70%	28.87%	42.84%
9	4.46%	22.74%	23.86%	48.94%
10	3.64%	24.15%	22.48%	49.73%
11	5.28%	33.73%	24.50%	36.49%

**Table 5:** Four regions’ D results.

No.	D_m_	R^2^	D_c_	R^2^	D_t_	R^2^	D_g_	R^2^
1	2.5524	0.981	2.2990	0.999	2.3060	0.999	2.3706	0.999
2	2.5633	0.979	2.4663	0.999	3.0467	0.997	2.4770	0.999
3	2.9158	0.958	2.3838	0.999	2.2728	0.999	2.1000	0.999
4	2.7745	0.960	2.5289	0.999	2.5412	0.999	2.6073	0.911
5	2.6860	0.969	2.5459	0.999	2.5424	0.999	2.4542	0.999
6	2.7391	0.964	2.5223	0.999	2.5554	0.999	2.9626	0.999
7	2.6513	0.973	2.7723	0.999	2.7070	0.999	2.4058	0.999
8	2.8081	0.950	2.6059	0.998	2.4523	0.999	2.3425	0.999
9	2.7871	0.978	2.5476	0.999	2.4216	0.999	2.3751	0.999
10	2.8151	0.955	2.4972	0.999	2.4536	0.999	2.3452	0.999
11	2.6586	0.970	2.6028	0.999	2.5472	0.999	2.3346	0.994

## Data Availability

The original contributions presented in this study are included in the article. Further inquiries can be directed to the corresponding authors.
